# Editorial: Wearables for human-robot interaction and collaboration

**DOI:** 10.3389/frobt.2025.1753153

**Published:** 2025-12-09

**Authors:** Xin Zhang, Anany Dwivedi, Yuquan Leng, Minas Liarokapis, Gustavo J. G. Lahr

**Affiliations:** 1 School of Electrical and Mechanical Engineering, University of Portsmouth, Portsmouth, United Kingdom; 2 Artificial Intelligence Institute, Division of Health, Engineering, Computing and Science, University of Waikato, Hamilton, New Zealand; 3 School of Biomedical Engineering, Harbin Institute of Technology (Shenzhen), Shenzhen, China; 4 New Dexterity Research Group, Department of Mechanical and Mechatronics Engineering, The University of Auckland, Auckland, New Zealand; 5 New Dexterity Research Group, School of Mechanical Engineering, National Technical University of Athens, Athens, Greece; 6 Instituto Israelita de Ensino e Pesquisa, Hospital Israelita Albert Einstein, Sao Paulo, Brazil

**Keywords:** wearables, human-robot interaction, motion capture systems, exoskeletons, human-machine interfaces

## Introduction

**FIGURE 1:**
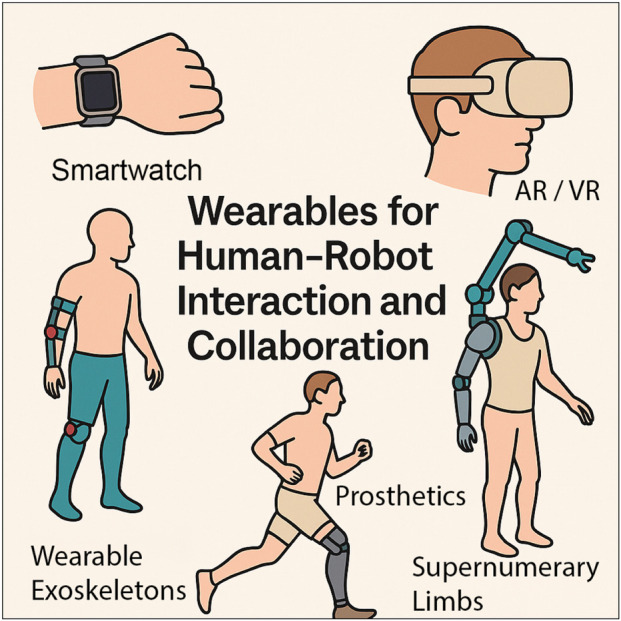
Wearables in people’s daily lives, ranging from upper and lower limbs exoskeletons to supernumerary limbs and augmented reality devices.

Wearable devices and related technologies have become deeply embedded in daily life. From consumer electronics to healthcare and industrial automation, wearables are now ubiquitous, enabling seamless interaction between humans and machines and fundamentally transforming modern lifestyles (Ates et al., 2022) as depicted in Figure 1. Devices such as smartwatches, intelligent armbands and gloves, smart insoles, and VR headsets are increasingly popular to support fitness, healthcare, and telepresence operations. At the same time, personalized wearable robots, including exoskeletons, prosthetic limbs, and supernumerary robotic arms, provide real-time physical support for rehabilitation, human motor restoration, and enhancement of capabilities. By inferring human intentions and interpreting behavioral and physiological signals, these systems are reshaping the foundations of human–robot interaction and collaboration. With the rise of concepts such as human-centered design (Boy, 2017; Wang et al., 2024) and Tri-cobots (coexisting–cooperative–cognitive robots) (Ding et al., 2018; Rahman et al., 2024), the development of wearable technologies has been accelerating.

This Research Topic has been launched to bring together the latest theoretical advancements and experimental progress across mechanical design, intelligent control and planning, machine learning algorithms, and innovative applications in the field of wearables for human-robot interaction. A total of eight manuscripts were submitted, of which four were accepted after a thorough peer-review process. The first two accepted articles focus on wearable sensing and related applications, while the latter two explore advancements in wearable robotics. A brief summary of the articles published is presented below.

## Article summary

Weigend et al. proposed a wearables-based motion capture (WearMoCap) solution that takes advantage of commercially available smartwatches and smartphones for robot control. In the WearMoCap system, smartwatches serve as the primary sensing device, while smartphones act as auxiliary modules. Unlike conventional motion capture systems that rely on optical tracking or dedicated inertial measurement unit (IMU) sensors, this smartwatch-based approach offers an innovative, low-cost alternative for partial-body pose tracking. WearMoCap supports three operating modes: (1) watch-only mode, which uses a single smartwatch; (2) upper arm mode, which combines a smartwatch and a smartphone worn on the upper arm; and (3) pocket mode, where the smartphone is placed in the user’s pocket. Given that both devices are equipped with a rich set of onboard sensors, such as gyroscopes, accelerometers, and barometric pressure sensors, the collected signals, together with arm length information, are used to regress upper-arm pose via supervised learning using ground-truth data from an optical motion capture system (OptiTrack). The pose estimation is performed using a long short-term memory (LSTM) network and a differentiable ensemble Kalman filter. The WearMoCap system was evaluated in four application scenarios: handover, intervention, teleoperation, and drone piloting. The results indicate that the watch-only mode provides the quickest setup, the upper-arm mode yields the highest accuracy, and the pocket mode offers the greatest flexibility with additional body orientation feedback. Furthermore, the authors released an open-source library of the WearMoCap.

Zhou et al. proposed a learning-based IMU state estimation approach to mitigate cumulative drift in manipulator teleoperation. IMU-driven teleoperation is prone to gyroscopic drift and accumulated error (up to 70° over 20 min), particularly in environments with magnetic interference where magnetometer calibration becomes unreliable. To address this Research Topic, the authors combined particle swarm optimization (PSO) for hyperparameter tuning with a modulated LSTM (ML-LSTM) for time-series error correction. A human-to-robot motion mapping experiment demonstrated that the proposed method reduced upper-arm orientation error from 30° to 10° and decreased end-effector position error from 0.25 m to 0.13 m. The results confirm that the PSO-ML-LSTM framework effectively suppresses IMU drift and enhances teleoperation accuracy.

Jenks et al. developed an open-source series elastic actuator (OpenSEA) for compliant human–robot interaction in exoskeleton joints. Based on Hooke’s Law, the SEA module leverages series elasticity to absorb impact forces and return stored energy to the output, which is the most core component for the whole exoskeleton robot. As SEAs contribute significantly to both performance and cost in exoskeletons, this article presents their design features in detail. The authors developed a fully 3D-printed OpenSEA module that integrates a planetary gear system and a torsional spring, achieving high torque density, compliance, and adaptability for elbow-rehabilitation applications. Experimental validation demonstrated a total compliance of 22.22°. Notably, the complete elbow exoskeleton was produced at a total cost of only $75.69, substantially lowering the barrier for accessible research and deployment in robotic rehabilitation.

Zhang et al. focused on the control of a SEA-based lower limb exoskeleton, and they proposed a sliding-mode control method based on prescribed performance control (PPC) for exoskeleton joints. The proposed controller ensures tracking errors remain within predefined convergence zones while addressing the limitations of fixed controller structures through a two-part design: a linear feedback component for system stabilization and a nonlinear component to maintain prescribed performance boundaries. The main innovations include modifying the controller denominator with a penalty constant to adjust transient characteristics (smoothness and response speed) and implementing an incremental PID controller combined with dynamics-based PPC for the exoskeleton. Simulations and experiments demonstrate the effectiveness of the proposed controller in maintaining constrained tracking errors while improving adaptability to modeling inaccuracies and system nonlinearities.

## Closing remarks

The contributions gathered in this Research Topic illustrate the diversity of current investigations in wearable technologies for human-robot interaction, covering sensing, learning, mechanical design, and control. Together, they demonstrate how advances in low-cost sensing, intelligent modeling, and compliant actuation are enabling the development of wearable systems that are functional, adaptable, and focused on human needs. As these technologies become more integrated into daily life, future research should continue to address interoperability, safety, and ethical considerations in human–machine interaction. It is hoped that this Research Topic will encourage further interdisciplinary studies and advance wearable intelligence for human assistance and rehabilitation.
